# Non-adherence to cardiometabolic medication as assessed by LC-MS/MS in urine and its association with kidney and cardiovascular outcomes in type 2 diabetes mellitus

**DOI:** 10.1007/s00125-024-06149-w

**Published:** 2024-04-22

**Authors:** Sara Denicolò, Vera Reinstadler, Felix Keller, Stefanie Thöni, Susanne Eder, Hiddo J. L. Heerspink, László Rosivall, Andrzej Wiecek, Patrick B. Mark, Paul Perco, Johannes Leierer, Andreas Kronbichler, Herbert Oberacher, Gert Mayer

**Affiliations:** 1 Department of Internal Medicine IV (Nephrology and Hypertension), Medical University Innsbruck, Innsbruck, Austria; 2 Institute of Legal Medicine and Core Facility Metabolomics, Medical University Innsbruck, Innsbruck, Austria; 3 Department of Clinical Pharmacy and Pharmacology, University of Groningen, University Medical Center Groningen, Groningen, the Netherlands; 4 International Nephrology Research and Training Center, Institute of Translational Medicine, Semmelweis University, Budapest, Hungary; 5 Department of Nephrology, Transplantation and Internal Medicine, Medical University of Silesia, Katowice, Poland; 6 School of Cardiovascular and Metabolic Health, University of Glasgow, Glasgow, UK

**Keywords:** Adherence, Cardiovascular disease, Diabetic kidney disease, Type 2 diabetes mellitus

## Abstract

**Aims/hypothesis:**

Non-adherence to medication is a frequent barrier in the treatment of patients with type 2 diabetes mellitus, potentially limiting the effectiveness of evidence-based treatments. Previous studies have mostly relied on indirect adherence measures to analyse outcomes based on adherence. The aim of this study was to use LC-MS/MS in urine—a non-invasive, direct and objective measure—to assess non-adherence to cardiometabolic drugs and analyse its association with kidney and cardiovascular outcomes.

**Methods:**

This cohort study includes 1125 participants from the PROVALID study, which follows patients with type 2 diabetes mellitus at the primary care level. Baseline urine samples were tested for 79 cardiometabolic drugs and metabolites thereof via LC-MS/MS. An individual was classified as totally adherent if markers for all drugs were detected, partially non-adherent when at least one marker for one drug was detected, and totally non-adherent if no markers for any drugs were detected. Non-adherence was then analysed in the context of cardiovascular (composite of myocardial infarction, stroke and cardiovascular death) and kidney (composite of sustained 40% decline in eGFR, sustained progression of albuminuria, kidney replacement therapy and death from kidney failure) outcomes.

**Results:**

Of the participants, 56.3% were totally adherent, 42.0% were partially non-adherent, and 1.7% were totally non-adherent to screened cardiometabolic drugs. Adherence was highest to antiplatelet and glucose-lowering agents and lowest to lipid-lowering agents. Over a median (IQR) follow-up time of 5.10 (4.12–6.12) years, worse cardiovascular outcomes were observed with non-adherence to antiplatelet drugs (HR 10.13 [95% CI 3.06, 33.56]) and worse kidney outcomes were observed with non-adherence to antihypertensive drugs (HR 1.98 [95% CI 1.37, 2.86]).

**Conclusions/interpretation:**

This analysis shows that non-adherence to cardiometabolic drug regimens is common in type 2 diabetes mellitus and negatively affects kidney and cardiovascular outcomes.

**Graphical Abstract:**

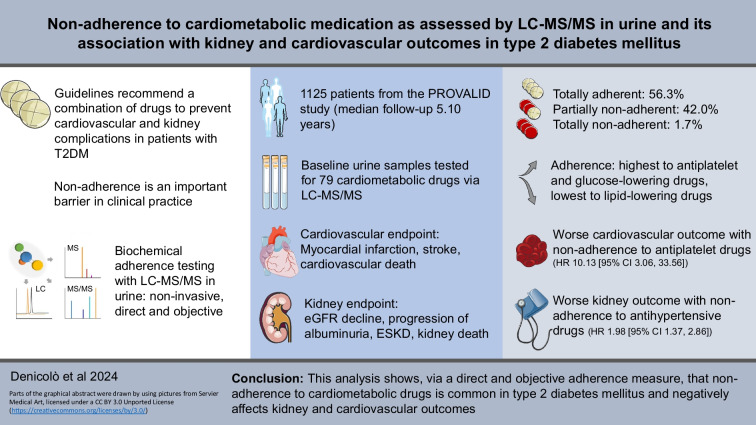

**Supplementary Information:**

The online version of this article (10.1007/s00125-024-06149-w) contains peer-reviewed but unedited supplementary material.


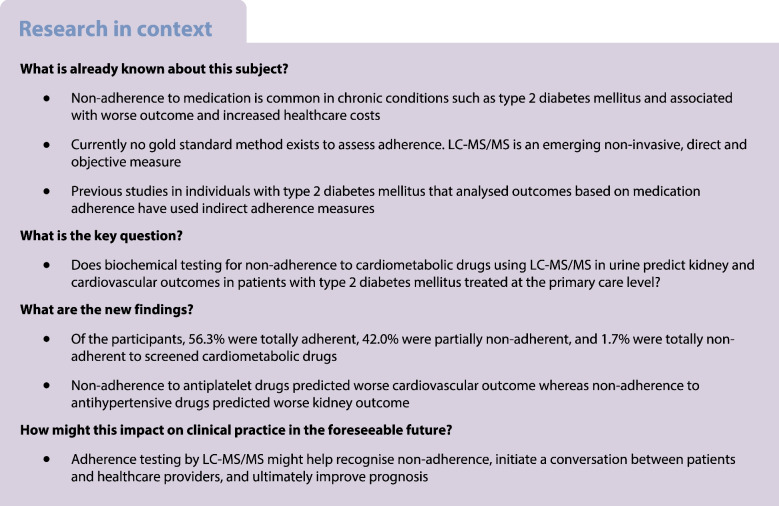


## Introduction

Cardiovascular and kidney complications contribute to excess morbidity and mortality in individuals with type 2 diabetes mellitus [1]. Besides lifestyle interventions, current guidelines recommend a combination of drugs to prevent or delay the incidence and progression of cardiovascular and kidney complications [2, 3]. Non-adherence to medication, however, is common when treating patients with type 2 diabetes mellitus [4].

Hitherto, epidemiological research has mostly relied on indirect adherence measures such as prescription refill data or questionnaires, with inherent limitations [5, 6]. Furthermore, most studies have focused on a single drug class [5].

Biochemical adherence testing in urine via LC-MS/MS is an emerging non-invasive, direct and objective measure to assess drug intake [6]. This method has previously been used in cross-sectional studies to estimate the prevalence of non-adherence to cardiometabolic drugs in populations with type 2 diabetes mellitus and to analyse factors associated with non-adherence [7, 8]. However, longitudinal studies also reporting the effect on outcome are currently lacking.

In this study, we apply this method to analyse non-adherence to different cardiometabolic drugs in a multinational cohort of patients with type 2 diabetes mellitus treated at the primary care level. We analyse factors associated with non-adherence and especially assess whether non-adherence influences cardiovascular and kidney outcomes. To the best of our knowledge, this is the first study to use a direct adherence measure to analyse how non-adherence to a wide spectrum of cardiometabolic drugs affects longitudinal outcome in type 2 diabetes mellitus.

## Methods

### Study population

**Fig. 1:**
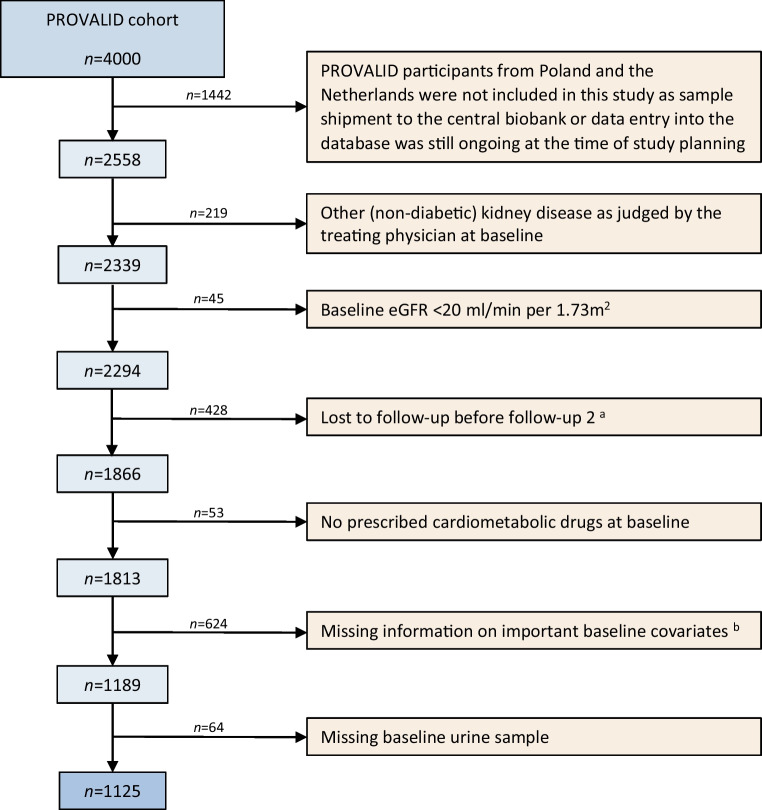
Selection of the study cohort. Inclusion criteria of PROVALID were type 2 diabetes mellitus with or without chronic kidney disease and age >18 years. The only exclusion criterion of PROVALID was malignancy requiring active treatment. ^a^As at least two follow-ups were needed to ascertain the UACR and eGFR endpoints, we excluded participants that were lost to follow-up before follow-up 2. Patients who reached a hard endpoint (KRT, or cardiovascular or kidney death) before follow-up 2, however, were included. ^b^Baseline covariates where no missing values were accepted: age, gender, BMI, smoking, year of diagnosis of type 2 diabetes mellitus, insulin use, year of diagnosis of hypertension, history of malignancy, history of diabetic retinopathy, history of heart failure, history of cerebrovascular disease, history of coronary artery disease, history of peripheral artery disease, kidney function (eGFR and albuminuria), LDL-cholesterol, systolic BP, HbA_1c_

PROVALID (Prospective Cohort Study in Patients with Type 2 Diabetes Mellitus for Validation of Biomarkers) is a multinational prospective observational cohort study of adults with type 2 diabetes mellitus recruited and followed annually at the primary healthcare level in five different European countries. Individuals were recruited between 2011 and 2014 and followed for a minimum of 4 years. Fasting morning blood and spot urine samples were collected at every annual study visit and stored in a central biobank at −80°C. PROVALID was observational and study participation did not affect patient management. Almost all patients included in the PROVALID study (98.9 %) were white. Gender was determined by self-report. The detailed study design and baseline characteristics of PROVALID are published elsewhere [9]. A total of 1125 eligible PROVALID participants were included in the present study. The selection process (including inclusion/exclusion criteria) is shown in Fig. 1. Baseline covariates for adjustment, including gender, were prespecified. Baseline characteristics of included and excluded individuals are compared in electronic supplementary material (ESM) Table 1. Baseline urine samples were used for LC-MS/MS measurements. The PROVALID study protocol was approved in each participating country by the responsible local institutional review boards. Signing an informed consent form was a prerequisite for study participation in all countries. This specific analysis was further approved by the ethics committee of the Medical University Innsbruck (EK Nr. 1235/2019).

### Study objectives and definitions

The primary objectives of this study were to assess the prevalence of non-adherence to cardiometabolic drugs in individuals with type 2 diabetes mellitus using a direct and objective adherence measure and to analyse non-adherence in the context of kidney and cardiovascular outcomes. We furthermore identified factors associated with non-adherence.

An individual was classified as totally adherent when markers for all analysed drugs were detected, partially non-adherent when at least a marker for one drug was detected and totally non-adherent when no marker for any prescribed drug was detected. This definition has been previously used in other studies [7, 8, 10–12].

We prespecified a cardiovascular and kidney composite endpoint for outcome analyses, respectively. The components of the cardiovascular endpoint were the classical 3-point major adverse cardiovascular events (MACE) defined by: (1) non-fatal stroke; (2) non-fatal myocardial infarction; and (3) cardiovascular death. The components of the kidney endpoint were defined as: (1) a sustained 40% reduction in eGFR (if baseline eGFR was >60 ml/min per 1.73m^2^, eGFR had to additionally fall below 60 ml/min per 1.73m^2^); (2) a sustained progression of albuminuria from normal/mildly increased albuminuria (urinary albumin/creatinine ratio [UACR] <30 mg/g creatinine) to moderately increased (UACR 30–300 mg/g creatinine) or severely increased albuminuria (UACR >300 mg/g creatinine) or from moderately increased albuminuria to severely increased albuminuria including a >30% increase in the mean UACR from baseline; (3) the initiation of kidney replacement therapy (KRT, dialysis or transplantation); or (4) death from kidney failure. Both eGFR decline and progression of albuminuria had to be sustained over at least one year as confirmed on the next annual in-study measurement, in order to differentiate progression of diabetic kidney disease from acute kidney injury or transient increases in albuminuria. The components of the kidney composite endpoint were selected based on the proposed definition of major adverse renal events (MARE) [13] and the international consensus definition of clinical trial outcomes for kidney failure [14]. eGFR was calculated using the Modification of Diet in Renal Disease equation [15], as prespecified in the PROVALID protocol. For the diagnosis of albuminuria, successive urinary albumin and creatinine measurements at three different time points were used to calculate UACR (mg/g). Albuminuria was classified based on a two-out-of-three principle (e.g. ‘normal to mildly increased albuminuria’, ‘normal to mildly increased albuminuria’, and ‘moderately increased albuminuria’ was classified as ‘normal to mildly increased albuminuria’). When fewer than three urine collections were available, albuminuria was determined by calculating the arithmetic mean. Non-fatal stroke, non-fatal myocardial infarction, cardiovascular death, initiation of KRT and death from kidney failure were investigator-reported.

### Sample preparation

Baseline urine samples were prepared by solid-phase extraction (SPE, Strata-X Microelution, 96-well plate, 2 mg/well, Phenomenex [Danaher Life Sciences, USA]). After adding fencamfamine as internal standard at a concentration of 60 ng/ml, a volume of 100 µl urine was processed according to the manufacture instructions. The obtained eluate (50 µl, 2% formic acid in acetonitrile) was diluted with 50 µl of water containing 1% heptafluorobutyric acid before LC-MS/MS analysis.

### Non-targeted LC-MS/MS

The LC-MS/MS system consisted of a Waters ACQUITY UPLC (Waters, Manchester, UK) coupled to a TripleTOF 5600+ (Sciex, Toronto, Canada). Chromatographic separations were performed on a Kinetex Biphenyl column (100 mm × 2.1 mm, 2.6 µm, 100 Å, Phenomenex), protected by a SecurityGuard ULTRA cartridge (UHPLC C18, 2.1 mm, Phenomenex) using a 15 min linear gradient of 2–98% acetonitrile in aqueous 0.5% acetic acid solution (vol./vol.). Sample aliquots of 7.5 µl were injected in ‘partial loop overfill’ mode. The temperature was held at 50°C and the flow rate was 200 µl/min. The mass spectrometer was operated in positive electrospray ionisation mode. The spray voltage was set to 5.5 kV. Gas flows of 50 arbitrary units for the nebuliser gas and 30 arbitrary units for the turbo gas were employed. The temperature of the turbo gas was adjusted to 400°C. The instrument was operated at an approximate mass resolving power of 30,000 for MS and 15,000 for MS/MS, and automatically recalibrated every ten sample injections using APCI positive calibration solution delivered via a calibration delivery system (Sciex). The scan range was mass-to-charge (*m/z*) 100–700 for MS, and *m/z* 50–700 for MS/MS. A duty cycle in the data-dependent acquisition mode included a single MS scan (accumulation time, 100 ms) followed by eight dependent MS/MS scans (accumulation time, 100 ms each) in the high sensitivity mode with dynamic background subtraction. MS/MS spectra were acquired at 35 eV with a collision energy spread of 10 eV. Former target ions were excluded for 30 seconds after two occurrences.

### Compound annotation and confirmation of drug intake

The obtained mass spectrometric data consisted of three levels of information: the retention time, the *m/z* values measured by high-resolution MS, and fragmentation information acquired in MS/MS. Annotation of a mass spectrometric feature to a chemical entity involved the matching of acquired data to reference data.

An overview of the 186 targets used for the confirmation of 77 drug compounds is provided in ESM Table 2. The targets included the drug compounds as well as human metabolites thereof. Information on the biotransformation of the investigated drugs was taken from literature. The *m/z* values were calculated by using the molecular formulas as input. Retention times and tandem mass spectra were obtained from analysing reference standards as well as urine samples of patients with confirmed drug consumption.

The acquired MS/MS spectra were extracted from raw data using MSConvert from ProteoWizard [16] and converted to plain text (ASCII) files with a program written in ActivePerl 5.6.1 (Active State Corporation, Vancouver, Canada). Automated library search of MS and MS/MS data was accomplished with ‘MSforID Search’ using the following settings: *m/z* tolerance ±0.01, intensity threshold value 0.01. A detailed description of the working principle of the search algorithm can be found elsewhere [17]. The proposed annotations were verified by the operator. The final output of data processing was a list of annotated targets and confirmed drug compounds.

### Quality assurance and quality control

Validation of the analytical workflow included the assessment of recoveries, matrix effects, limits of identification and selectivity with commercially available reference standards of 52 targets. Positive control samples included mixtures of reference standards spiked into blank urine samples at different concentrations. Negative control samples included different kinds of blank samples (procedural and solvent blanks). The experiments proved fitness of the developed workflow for adherence testing. Targets were detectable down to the low ng/ml range. No false-positive results were produced.

Quality control involved the repeated analysis of a reference sample containing 40 compounds of interest at a concentration of 10 ng/ml each. Overall, 108 quality control samples were analysed together with the study samples. Within a sample batch, the errors of the measured *m/z* values were within ±10 ppm, standard deviations of the retention times were below 3 s, and the standard deviations of the peak areas were smaller than 25%. Moreover, no false-positive results were produced by analysing negative control samples.

### Detection of drugs and drug metabolites

A total of 91 different cardiometabolic drugs—including glucose-lowering, antihypertensive, lipid-lowering and antiplatelet drugs as well as diuretics—were prescribed to included patients at baseline (Table 1). Out of these 91 drugs, 77 were detectable in urine samples by LC-MS/MS (Table 1). Twelve drugs could not be detected, primarily due to drug-related or methodological issues (Table 1, ESM Table 3). Nimodipine and spirapril were each prescribed to only one single participant and not detected. As there was no clear rationale for excluding these drugs, these two individuals were classified as non-adherent to nimodipine und spirapril, respectively. In the event that another drug or associated metabolite from the same drug subclass was detected instead of the drug listed in the baseline medication list (e.g. nebivolol instead of bisoprolol or lisinopril instead of ramipril), the patient was considered to be adherent as a recent change in prescription seemed most likely (observed in 118 of 1125 participants). This may occur when patients are being treated by different specialists, which is not unusual for people with type 2 diabetes mellitus, and when drug availabilities in pharmacies play a role.

**Table 1:** Prescribed cardiometabolic medications

Antihypertensive drugs	Glucose-lowering drugs	Diuretics	Lipid-lowering drugs	Antiplatelet drugs
Amlodipine	Metformin	Torasemide	Atorvastatin	Clopidogrel
Lercanidipine	Linagliptin	Hydrochlorothiazide	Rosuvastatin	Ticlopidine
Nitrendipine	Vildagliptin	Chlortalidone	Simvastatin	Acetylsalicylic acid^a^
Felodipine	Sitagliptin	Xipamide	Fluvastatin	Ticagrelor^a^
Nifedipine	Saxagliptin	Indapamide	Ezetimibe	Eptifibatide^a^
Nimodipine	Pioglitazone	Bendroflumethiazide	Bezafibrate	
Valsartan	Glibenclamide	Clopamide	Ciprofibrate	
Losartan	Gliclazide	Spironolactone	Fenofibrate	
Candesartan	Glimepiride	Eplerenone	Pravastatin^a^	
Irbesartan	Glipizide	Amiloride		
Olmesartan	Gliquidone	Triamterene		
Eprosartan	Nateglinide	Furosemide^a^		
Telmisartan	Repaglinide	Bumetanide^a^		
Ramipril	Liraglutide^a^	Butizide^a^		
Lisinopril	Exenatide^a^			
Enalapril	Acarbose^a^			
Captopril				
Cilazapril				
Fosinopril				
Benazepril				
Imidapril				
Perindopril				
Quinapril				
Spirapril				
Trandolapril				
Carvedilol				
Bisoprolol				
Metoprolol				
Nebivolol				
Atenolol				
Betaxolol				
Pindolol				
Propanolol				
Sotalol				
Doxazosin				
Urapidil				
Alfuzosin				
Prazosin				
Rilmenidine				
Moxonidine				
Verapamil				
Diltiazem				
Nicorandil				
Dipyridamole				
Aliskiren				
Lacidipine^a^				
Hydralazine^a^				

^a^Drugs that could not be analysed mainly due to drug-related or methodological issues (more details regarding these drugs are provided in ESM Table 3). All other listed drugs were detectable by LC-MS/MS

### Statistical analysis

Patient characteristics are described with absolute and relative frequencies for discrete variables, and mean and SD or, when appropriate, e.g. due to skewness, median and 1st and 3rd quartile for continuous variables. Hypotheses of no differences in scale or distribution of patient characteristics between groups were tested with *t* tests, or when appropriate, with Wilcoxon–Mann–Whitney tests for continuous and with χ^2^ homogeneity tests for categorical variables. There were no missing values in the patient characteristics. Adherence shares in participants grouped by drug class, age, disease duration and number of drugs are sample averages and presented with 95% CI. Based on these groups heterogeneity in adherence distribution was assessed. For two-dimensional contingencies, the *χ*^2^ test was employed, while for three-dimensional contingencies, the Cochran–Mantel–Haenszel test was used. The distributions of HbA_1c_, LDL-cholesterol, systolic BP and UACR split by adherence in drug groups are displayed with boxplots. Notches around the median indicate 95% CI. *p* values for group differences correspond to the null hypothesis of Wilcoxon–Mann–Whitney tests. Cox proportional hazard regression models were performed to estimate HR of the effect of non-adherence on the above defined endpoints. To account for potential country-specific heterogeneity regarding the baseline hazards we performed a country-wise stratified Cox regression for each endpoint. With the intention to address potential confounding, models were additionally adjusted for baseline variables (age, gender, smoking status, diabetes duration, systolic BP, eGFR, UACR, BMI, LDL-cholesterol, HbA_1c_, heart failure and atherosclerotic cardiovascular disease). Atherosclerotic cardiovascular disease comprises cerebrovascular disease, coronary artery disease and peripheral artery disease. All adjustment variables were normalised to mean 0 and SD 1. Estimates are presented with 95% CIs. We allowed for a type 1 error of 5%, with all hypotheses being two-sided. All analyses were carried out using R 4.2.2 [18].

## Results

### Characteristics of the study population and prevalence of non-adherence

The data of 1125 eligible patients was analysed. The median (IQR) follow-up time was 5.10 (4.12–6.12) years. The median age was 65.00 (59.00–70.00) years and 46.1% were women. The median duration of type 2 diabetes mellitus was 10.00 (5.00–15.00) years. The mean (±SD) HbA_1c_ was 53.66±12.24 mmol/mol (7.06±1.12%), the mean eGFR was 77.55±23.64 ml/min per 1.73 m^2^ and median albuminuria 10.54 (5.16–26.49) mg/g creatinine. Full baseline characteristics of the study population are shown in Table 2 and compared between adherent and non-adherent individuals.

**Table 2:** Baseline characteristics

Variable	Total cohort *n*=1125	Totally or partially non-adherent *n*=492	Totally adherent *n*=633
Age, years	65.00 [59.00–70.00]	65.00 [59.00–70.00]	65.00 [59.00–70.00]
Gender (%)			
Women	519 (46.1)	230 (46.7)	289 (45.7)
Men	606 (53.9)	262 (53.3)	344 (54.3)
BMI, kg/m^2^	31.02 [28.19–34.68]	30.84 [28.05–34.48]	31.22 [28.37–34.96]
Systolic BP, mmHg	137.37 (16.63)	138.68 (16.79)	136.36 (16.44)
Smoking (%)			
Never	572 (50.8)	263 (53.5)	309 (48.8)
Current or ex-smoker	553 (49.2)	229 (46.5)	324 (51.2)
History of malignancy (%)	61 (5.4)	29 (5.9)	32 (5.1)
Heart failure (%)	39 (3.5)	17 (3.5)	22 (3.5)
Coronary artery disease (%)	229 (20.4)	105 (21.3)	124 (19.6)
Peripheral artery disease (%)	107 (9.5)	56 (11.4)	51 (8.1)
Cerebral artery disease (%)	83 (7.4)	38 (7.7)	45 (7.1)
Duration of hypertension, years	12.00 [7.00–19.00]	12.00 [6.00–20.00]	12.00 [7.00–18.00]
Duration of T2DM, years	10.00 [5.00–15.00]	11.00 [6.00–16.25]	10.00 [5.00–14.00]
Diabetic retinopathy (%)			
Yes	209 (18.6)	101 (20.5)	108 (17.1)
No	823 (73.2)	354 (72.0)	469 (74.1)
Unknown	93 (8.3)	37 (7.5)	56 (8.8)
Insulin therapy (%)	342 (30.4)	164 (33.3)	178 (28.1)
eGFR, ml/min per 1.73 m^2^	77.55 (23.64)	78.46 (25.23)	76.84 (22.32)
Albuminuria category (%)			
Normal to mildly increased albuminuria	890 (79.1)	380 (77.2)	510 (80.6)
Moderately increased albuminuria	186 (16.5)	85 (17.3)	101 (16.0)
Severely increased albuminuria	49 (4.4)	27 (5.5)	22 (3.5)
Albuminuria, mg/g	10.54 [5.16–26.49]	11.16 [5.63–29.61]	9.83 [4.83–24.53]
HbA_1c,_ mmol/mol	53.66 (12.24)	53.88 (12.46)	53.55 (12.02)
HbA_1c_, %	7.06 (1.12)	7.08 (1.14)	7.05 (1.10)
LDL-C, mmol/l	2.56 (0.98)	2.69 (1.01)	2.46 (0.94)
Number of prescribed drugs	5.39 (2.03)	5.75 (2.12)	5.12 (1.92)
Number of screened drugs	4.83 (1.78)	5.15 (1.85)	4.58 (1.69)
Number of detected drugs	4.16 (1.82)	3.63 (1.85)	4.58 (1.69)

Discrete variables are shown as absolute and relative frequencies, *n* (%), and continuous variables as mean (SD) or, when appropriate, e.g. due to skewness, median [IQR]

LDL-C, LDL-cholesterol; T2DM, type 2 diabetes mellitus

**Fig. 2:**
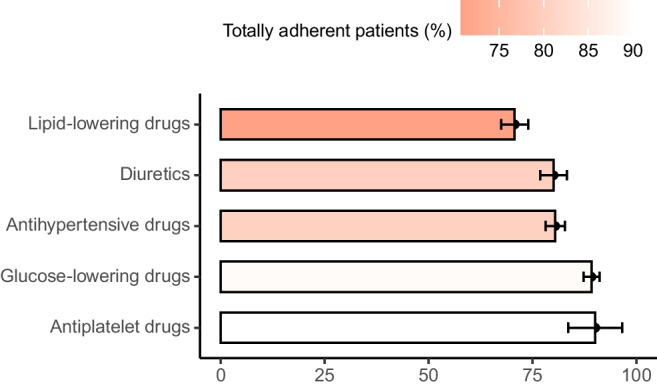
Adherence by drug class. Adherence was found to be highest to antiplatelet and glucose-lowering drugs and lowest to lipid-lowering drugs. Bars represent sample averages and whiskers 95% CIs

Participants were prescribed 5.39 (±2.03) cardiometabolic drugs on average (mean ± SD) at baseline. Of these, an average (mean ± SD) of 4.83 (±1.78) could be screened by LC-MS/MS in baseline urine samples. Based on the results of LC-MS/MS measurements, 633 (56.3%) participants were totally adherent, 472 (42.0%) were partially non-adherent and 20 (1.7%) were totally non-adherent to all screened cardiometabolic drugs. Adherence was highest to antiplatelet and glucose-lowering drugs and lowest to lipid-lowering drugs (Fig. 2). Adherence by subclasses (e.g. ACE inhibitors, beta blockers, metformin etc.) is provided in ESM Table 4. Participants who were adherent to lipid-lowering drugs had a significantly lower LDL-cholesterol (median [IQR] totally adherent: 2.06 [1.63–2.58] mmol/l vs totally or partially non-adherent: 2.80 [2.10–3.54] mmol/l, *p*<0.001) and those who were adherent to antihypertensive drugs had a significantly lower UACR (median totally adherent: 9.87 [4.88–25.39] mg/g vs totally or partially non-adherent: 12.55 [6.53–44.51] mg/g, *p*=0.002). A trend for lower HbA_1c_ with adherence to glucose-lowering drugs (median totally adherent: 51.04 [45.36–59.56] mmol/mol vs totally or partially non-adherent: 54.10 [45.68–60.66] mmol/mol, *p*=0.325) and lower systolic BP (median totally adherent:135.00 [125.00–145.00] mmHg vs totally or partially non-adherent: 140.00 [130.00–149.00] mmHg, *p*=0.091) with adherence to antihypertensive drugs could be observed (ESM Fig. 1).

### Factors associated with non-adherence

**Fig. 3:**
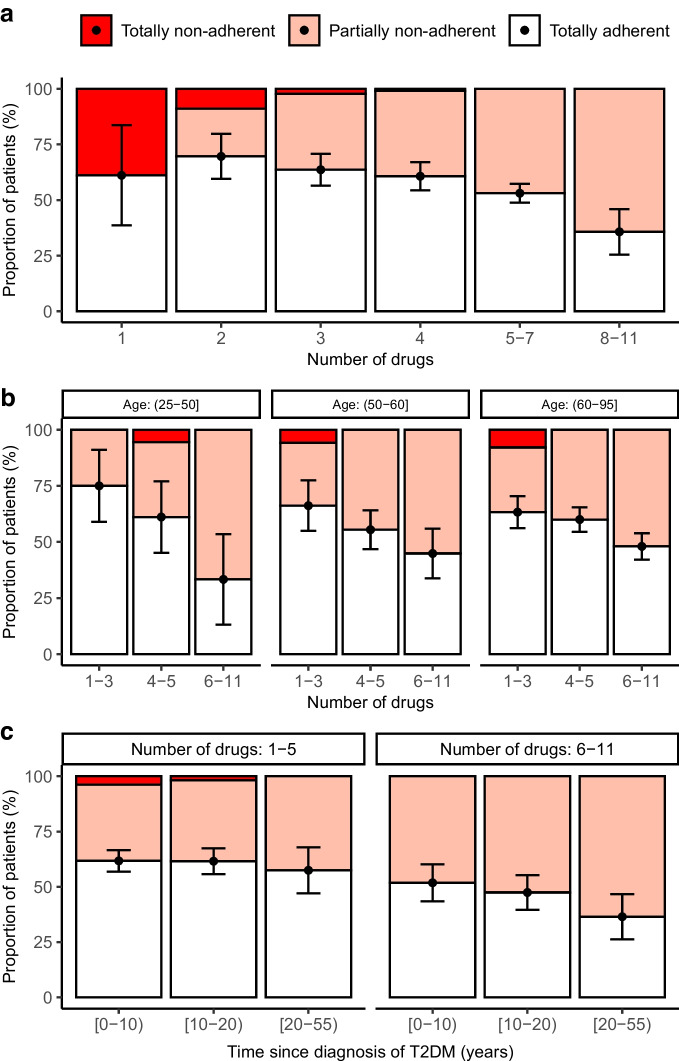
Adherence by number of drugs, age and duration of T2DM. Bars indicate proportions of totally non-adherent, partially non-adherent and totally adherent patients with 95% CIs for totally adherent patients. ‘(’ or ‘)’ indicates that the number is excluded from the range and ‘[’ or ‘]’ indicates that the number is included. (**a**) Adherence subdivided by the number of drugs. χ^2^ test for heterogeneity: χ^2^=215.66, *df*=10, *p*<0.001. (**b**) Relationship between adherence, age and number of drugs. Cochran–Mantel–Haenszel test for heterogeneity: M^2^ =31.092, *df*=2, *p*<0.001. (**c**) Adherence by duration of T2DM and number of drugs. Cochran–Mantel–Haenszel test for heterogeneity: M^2^ = 24.684, *df* = 2, *p*<0.001. T2DM, type 2 diabetes mellitus

Participants who were totally and partially non-adherent had a higher number (mean ± SD) of cardiometabolic drugs as compared with those who were totally adherent (5.75±2.12 vs 5.12±1.92, *p*<0.001). In particular, partial non-adherence increased with an increasing number of drugs (Fig. 3a). Age per se had no influence on adherence, however the number of drugs had more impact on adherence in younger patients (Fig. 3b). Non-adherent individuals had a longer (median [IQR]) history of type 2 diabetes mellitus (11.00 [6.00–16.25] years vs 10.00 [5.00–14.00] years, *p*=0.011), which was also influenced by the number of drugs (Fig. 3c). Ex-smokers were more likely to be adherent than current or never-smokers (OR 1.4, *p*=0.014). No difference in adherence between women and men could be detected (55.7% of women and 56.8% of men were totally adherent). Adherence by drug class also did not significantly differ between women and men (ESM Fig. 2).

### Association of non-adherence with cardiovascular and kidney outcomes

Over a median (IQR) follow-up time of 5.10 (4.12–6.12) years, 161 (14.3%) participants suffered a kidney event (progression of albuminuria: 127, decline of eGFR: 27, KRT: 11, death due to kidney failure: 4) and 94 (8.4%) participants had a cardiovascular event (stroke: 38, myocardial infarction: 33, cardiovascular death: 34). Eight patients suffered more than one kidney event, and 11 suffered more than one cardiovascular event. In those cases, the first event was used for the composite analysis.

**Fig. 4:**
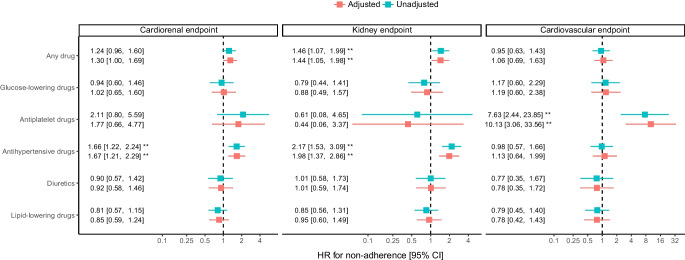
Association of non-adherence with cardiovascular and kidney outcomes. Relative risks are shown as HR with 95% CI. Adjusted analyses are indicated in red and unadjusted analyses in blue. Adjustment variables were baseline age, gender, BMI, smoking, heart failure, history of atherosclerotic cardiovascular disease, eGFR, albuminuria, BP, HbA_1c_, LDL-cholesterol and duration of type 2 diabetes mellitus. Analyses were further stratified by country

In the longitudinal analysis, non-adherence in general (to any cardiometabolic drug) was associated with increased kidney but not cardiovascular events. This was mostly driven by the effect of non-adherence to antihypertensive drugs on the kidney endpoint (HR 1.98 [95% CI 1.37, 2.86]). Worse cardiovascular outcome was observed with non-adherence to antiplatelet drugs (HR 10.13 [95% CI 3.06, 33.56]) (Fig. 4). Analyses were stratified by country and adjusted for baseline age, gender, BMI, smoking, heart failure, atherosclerotic cardiovascular disease, eGFR, albuminuria, BP, HbA_1c_, LDL-cholesterol and duration of type 2 diabetes mellitus. Absolute numbers of patients on each drug class, absolute numbers of events as well as incidence rates of events are shown in ESM Table 5. ESM Table 6 shows absolute numbers of specific cardiovascular and kidney events (components of composite endpoints) as well as incidence rates by adherence to drug classes.

## Discussion

In this study, we found a high prevalence (42.0%) of partial non-adherence to cardiometabolic medications, i.e. patients taking some but not all prescribed drugs. Total non-adherence was rare (1.7%). Adherence testing by urine LC-MS/MS has previously been used in cross-sectional studies of individuals with type 2 diabetes mellitus [7, 8]. Patel and colleagues [8] analysed adherence to cardiometabolic drugs in 228 people with type 2 diabetes mellitus treated at the primary care level in the UK. Using the same definition of adherence, they found 22.4% to be partially and 5.7% to be totally non-adherent. The lower non-adherence rate might be partially explained by younger age and fewer prescribed/screened cardiovascular medications. Similar to our results, adherence was lowest to lipid-lowering drugs. The authors speculate that side effects such as myalgia or the negative perception of statins in the general population might explain this observation [8, 19]. Adherence to antiplatelet drugs was not analysed in their study. Beernink and colleagues [7] analysed adherence to glucose-lowering and antihypertensive drugs as well as statins in 457 individuals included in the DIALECT study. The study was conducted at the specialist care level in the Netherlands. They found 10.7% of participants to be non-adherent. The authors speculate that the high adherence rate may be explained by the specialist care setting and the well-organised pharmacy service in the Netherlands, where medication is often delivered automatically [7]. Micro- and macrovascular complications were more often observed in non-adherent participants; however, the analysis was only cross-sectional and the sample size not adequate to analyse adherence at the level of individual drug classes [7].

We found higher LDL-cholesterol levels in participants who were non-adherent to lipid-lowering drugs and a trend towards higher HbA_1c_ levels in those who were non-adherent to glucose-lowering drugs. Similar results were reported by Patel and colleagues [8]. The non-significant difference in HbA_1c_ may be explained by other factors influencing glycaemic control such as (correct) insulin use and lifestyle factors.

In our study, partial non-adherence in particular increased continuously as the number of drugs increased. This is in line with results of other studies [20]. Gupta and colleagues [21] showed that each increase in the number of antihypertensive medications led to a marked increase in non-adherence, and they argue that the number of drugs represents a modifiable risk factor. In our study, the association between number of drugs and adherence was particularly clear in younger patients. A longer diabetes duration was associated with lower adherence; however, this also seems to be explained, at least in part, by an increasing number of drugs with longer disease duration. Interestingly, the percentage of totally non-adherent participants decreased with an increasing number of drugs (as the percentage of partially non-adherent participants increased). Possible explanations might be that patients who only take one or two drugs are less advanced in their disease and thus may have a lower disease burden, and taking medications may be less integrated in their daily lives as compared with those on multiple medications. At the same time, the complexity of medication schedules increases as the number of drugs increases and it may be more challenging for patients to follow these schedules accurately. Participants might also feel that the burden of taking multiple medications is too high and choose to omit certain medications. However, these are only hypotheses as we did not have data available to inform on barriers and beliefs that could have led to total or partial non-adherence. Ex-smoking status was associated with adherence. We speculate that motivation and disease awareness that had led to smoking cessation may also have led to enhanced adherence behaviour.

Although acetylsalicylic acid, the most prescribed antiplatelet drug, could not be detected by our assay, non-adherence to antiplatelet drugs (clopidogrel prescribed second most frequently) was strongly associated with worse cardiovascular outcome. The observed effects of non-adherence to antihypertensive drugs on the cardiorenal endpoint and general non-adherence on the kidney endpoint are likely to be driven by the effect of non-adherence to antihypertensive drugs on the kidney endpoint. We did not find an association between non-adherence to antihypertensive drugs, lipid-lowering drugs and glucose-lowering drugs with cardiovascular outcome. Similarly, we did not find an association between non-adherence to glucose-lowering drugs with kidney outcome. As the median (IQR) follow-up time was 5.10 (4.12–6.12) years, it is possible that the observation period was too short to observe a significant effect.

In this and previous studies using this method, a qualitative approach (drug/metabolites are either present or not present) was used. Currently, qualitative screening is preferred over quantitative analyses as no clear cut-offs are established [6]. Urine collection and shipment are not likely to have compromised results as samples were stored at – 80°C and a study by Burns and colleagues [22] has shown that cardiometabolic drugs and drug metabolites are stable in urine even at room temperature for at least three days [22]. The same authors also found that urine concentration does not affect results [22, 23]. Further, biochemical adherence testing in urine has been shown to be also reliable in the setting of chronic kidney disease [24].

This study has several strengths. It analyses non-adherence to a wide range of cardiometabolic drugs using a non-invasive, direct and objective as well as sensitive and specific adherence measure. The study population is well characterised with available long-term follow-up and represents a real-world setting of individuals with type 2 diabetes mellitus treated at the primary care level.

However, this study also has several limitations. Although we were able to include 79 of 91 prescribed cardiometabolic drugs, 12 drugs were not detectable. In particular, the use of antiplatelet drugs is under-represented in this study, as acetylsalicylic acid (prescribed to 378 patients) was not detectable by LC-MS/MS due to a short half-life of the parent drug, complex metabolisation and endogenously occurring metabolites [24]. Clopidogrel was the most frequently prescribed antiplatelet agent that could be detected in this study. Glucagon-like peptide-1 (GLP-1) agonists were not detectable due to cleavage into non-detectable peptides by peptidases. Furosemide and bumetanide could also not be detected. This may be explained by a short half-life, preference of morning intake and samples collected in the morning from fasting participants. At the time of baseline visits, sodium–glucose cotransporter 2 (SGLT2) inhibitors were not yet as established in the treatment of people with diabetes and cardiorenal protection as they are now, thus none of the included participants had an SGLT2 inhibitor in their prescription and consequently we were not able to analyse adherence to SGLT2 inhibitors.

Currently, no consensus exists on a gold standard method for detecting non-adherence [25]. Despite being direct and objective, biochemical adherence testing by LC-MS/MS has limitations. In contrast to other adherence measures (e.g. prescription refill data or electronic monitoring) it only provides a snapshot of adherence. Thus, a single measurement (as performed in this study) does not inform on changes of adherence over time and also does not reliably detect irregular drug intake. However, a study by Gupta and colleagues also showed that a single assessment of non-adherence can predict adverse long-term outcomes in patients with heart failure [26]. In a study by Hamdidouche and colleagues, the adherence status remained stable in 88% of participants upon a second LC-MS/MS measurement after 11 months [11]. Although qualitative adherence testing by LC-MS/MS is a sensitive and specific method [6, 27, 28], it cannot be excluded that pharmacokinetic factors, genetic and pharmacogenetic characteristics, drug–drug interactions, diet, lifestyle factors and comorbidities affect the ability to detect drugs or metabolites in urine. Further, assessing adherence via LC-MS/MS does not provide information on barriers and beliefs that could have influenced non-adherence. We were not able to include a second adherence measure to add this additional level of information or to compare results as the necessary data were not collected within the PROVALID study. Adherence might have been overestimated due to white coat adherence/the ‘toothbrush effect’ (i.e. non-adherent patients take their medication before a doctor’s appointment) [29]. White coat adherence and the Hawthorne effect (i.e. people change their behaviour when they know that they are being studied) [30] due to study participation, however, are considered to be minimal. The participants, who gave informed consent for PROVALID participation and future studies on stored blood and urine samples, were not aware of adherence testing as this specific study was planned after sample collection was completed.

The effect of adherence on outcomes might have been overestimated due to the healthy adherer effect (i.e. participants who are adherent also tend to have healthier lifestyles) [31]. Although the analyses were adjusted for several potentially confounding variables indirectly moderated by lifestyle, we were not able to provide and directly adjust for lifestyle factors, other than BMI and smoking, as such variables were not assessed within the PROVALID study. Similar results in the adjusted and non-adjusted analyses and no evident effects of adherence to drug classes with no known direct benefit on a specific outcome (e.g. adherence to antiplatelet drugs on kidney outcome), however, suggest that the healthy adherer effect has not significantly biased results.

In conclusion, our LC-MS/MS analysis of spot urine samples directly showed that non-adherence to cardiometabolic drugs is common in individuals with type 2 diabetes mellitus treated at the primary care level. Non-adherence to antiplatelet drugs predicted worse cardiovascular outcome whereas non-adherence to antihypertensive drugs predicted worse kidney outcome. To the best of our knowledge, this study is the first to use a direct adherence measure to analyse the effect of adherence to different cardiometabolic drug classes on longitudinal cardiovascular and kidney outcomes in type 2 diabetes mellitus. Adherence testing by LC-MS/MS might help to recognise non-adherence, start a conversation between patients and healthcare providers and ultimately improve prognosis.

### Supplementary Information

Below is the link to the electronic supplementary material.

ESM1 (PDF 519 KB)

ESM2 (XLSX 55 KB)

## Data Availability

The datasets generated during and/or analysed in the current study are available from the corresponding author upon reasonable request and after discussion of the request within the PROVALID steering committee.

## Abbreviations

KRT: Kidney replacement therapy
m/z values: Mass-to-charge values
PROVALID: Prospective Cohort Study in Patients with Type 2 Diabetes Mellitus for Validation of Biomarkers
UACR: Urinary albumin/creatinine ratio
