# Supplementary material for: Non-adherence to cardiometabolic medication as assessed by LC-MS/MS in urine and its association with kidney and cardiovascular outcomes in type 2 diabetes mellitus
Source: Diabetologia. 2024 Apr 22;67(7):1283–94. doi: 10.1007/s00125-024-06149-w (PMC11153278; doi:10.1007/s00125-024-06149-w)
Supplement: Supplementary file 1 — ESM1 (PDF 519 KB) [file 125_2024_6149_MOESM1_ESM.pdf]

Non-adherence to cardiometabolic medication as assessed by LC-MS/MS in urine and its association with kidney and cardiovascular outcomes in type 2 diabetes mellitus

Supplementary Material

Table of content

ESM Table 1. Baseline characteristics of included and excluded PROVALID participants 2

ESM Table 2. Overview of drug targets 3

ESM Table 3. Excluded drugs 3

ESM Table 4. Adherence by drug subclasses 4

ESM Table 5. Absolute numbers of events and incidence rates by adherence to drug classes 5

ESM Table 6. Absolute numbers of specific cardiovascular and kidney events (components of composite endpoints) and incidence rates by adherence to drug classes 6

ESM Fig. 1. Comparison of target values by adherence to corresponding drug classes 8

ESM Fig. 2. Adherence to drug classes by gender 9

ESM Table 1. Baseline characteristics of included and excluded PROVALID participants

**Variable**	**Unit**	**Overall**	**Excluded**	**Included**
n		2558	1433	1125
Age (median [IQR])	years	64.00 [57.00–70.00]	63.00 [56.00–69.00]	65.00 [59.00–70.00]
Gender (%)	women	1133 (44.3)	614 (42.9)	519 (46.1)
	men	1424 (55.7)	818 (57.1)	606 (53.9)
BMI (median [IQR])	kg/m²	30.49 [27.48–34.34]	30.11 [26.93–34.02]	31.02 [28.19–34.68]
Systolic blood pressure (mean (SD))	mmHg	135.97 (16.71)	134.86 (16.71)	137.37 (16.63)
Smoking (%)	never	1289 (50.7)	717 (50.6)	572 (50.8)
	current or ex-smoker	1252 (49.3)	699 (49.4)	553 (49.2)
History of malignancy (%)		149 (5.8)	88 (6.2)	61 (5.4)
Heart failure (%)		79 (3.1)	40 (2.8)	39 (3.5)
Coronary artery disease (%)		523 (20.5)	294 (20.6)	229 (20.4)
Peripheral artery disease (%)		212 (8.3)	105 (7.4)	107 (9.5)
Cerebral artery disease (%)		203 (8.0)	120 (8.4)	83 (7.4)
Duration of hypertension (median [IQR])	years	12.00 [7.00–19.00]	12.00 [6.00–19.00]	12.00 [7.00–19.00]
Duration of T2DM (median [IQR])	years	9.00 [4.00–15.00]	8.00 [3.00–14.00]	10.00 [5.00–15.00]
Diabetic retinopathy (%)	yes	416 (16.3)	207 (14.6)	209 (18.6)
	no	1907 (74.9)	1084 (76.2)	823 (73.2)
	unknown	224 (8.8)	131 (9.2)	93 (8.3)
Insulin therapy (%)		743 (29.0)	401 (28.0)	342 (30.4)
eGFR (mean (SD))	ml/min/1.73m²	77.50 (24.58)	77.46 (25.33)	77.55 (23.64)
Albuminuria category (%)	normal to mildly increased albuminuria	1927 (78.2)	1037 (77.4)	890 (79.1)
	moderately increased albuminuria	430 (17.4)	244 (18.2)	186 (16.5)
	severely increased albuminuria	108 (4.4)	59 (4.4)	49 (4.4)
Albuminuria (median [IQR])	mg/g	9.93 [5.02–27.06]	9.58 [4.97–27.56]	10.54 [5.16–26.49]
HbA_{1c} (mean (SD))	mmol/mol	53.99 (13.99)	54.21 (15.30)	53.66 (12.24)
HbA_{1c} (mean (SD))	%	7.09 (1.28)	7.11 (1.40)	7.06 (1.12)
LDL (mean (SD))	mmol/l	2.67 (1.02)	2.79 (1.05)	2.56 (0.98)

ESM Table 1. Comparison between included and excluded PROVALID participants. Participants from Austria, Hungary and the UK (n = 2558) represented the basis for the selection process. Socioeconomic factors were not assessed within the PROVALID study. Discrete variables are shown as absolute and relative frequencies and continuous variables as mean and standard deviation or, when appropriate e.g. due to skewness, median and 1st and 3rd quartile. IQR, interquartile range; BMI, body mass index; SD, standard deviation; T2DM, type 2 diabetes mellitus; eGFR, estimated glomerular filtration rate; HbA_{1c}, haemoglobin A1c; LDL, low-density lipoprotein.

ESM Table 2. Overview of drug targets

Please refer to the excel file "ESM Table 2 - Overview of Targets.xlsx" for an overview of the targets used to verify drug consumption by LC-MS/MS

ESM Table 3. Excluded drugs

Drug	Prescribed	Detected	Rationale for exclusion
Acarbose	14	0	Drug-related issue
Acetylsalicylic acid	378	0	Drug-related issues
Bumetanide	1	0	Drug-related issues
Butizide	1	0	NA
Eptifibatide	1	0	Drug-related and methodological issues
Exenatide	2	0	Drug-related and methodological issues
Furosemide	142	0	Drug-related issues
Hydralazine	4	0	Drug-related issues
Lacidipine	3	0	Drug-related issues
Liraglutide	11	0	Drug-related and methodological issues
Pravastatin	6	0	Drug-related issues
Ticagrelor	25	0	Database issues

ESM Table 3. 12 drugs could not be detected in this study – mostly due to drug-related or methodological issues. Ticagrelor was excluded due to database ambiguity. Butizide was prescribed to one patient and not detected. Due to lacking information and rationales for inclusion, the drug was excluded.

ESM Table 4. Adherence by drug subclasses

Drug subclass	Prescribed	Adherent	% adherent
ACE inhibitors	618	552	89.3
Aldosterone antagonists	35	27	77.1
Alpha blockers	170	136	80.0
Angiotensin II receptor blockers	400	356	89.0
Beta blockers	603	559	92.7
Biguanides (metformin)	896	849	94.8
Calcium channel blockers	418	370	88.5
Centrally acting antihypertensives	97	66	68.0
Clofibric acid derivatives	92	71	77.2
DPP-4 inhibitors	273	242	88.6
Dipyridamole	7	7	100.0
Direct vasodilators	9	2	22.2
Loop diuretics	4	4	100.0
Meglitinides (glinides)	9	7	77.8
Others lipid lowering drugs (ezetimibe)	41	28	68.3
Potassium sparing diuretics	26	17	65.4
Renin inhibitors	3	3	100.0
Statins	715	513	71.8
Sulfonylureas	334	304	91.0
Thiazide diuretics	555	453	81.6
Thiazolidinediones (glitazones)	53	48	90.6
Thienopyridine derivatives	74	66	89.2

ESM Table 4. “Prescribed” indicates the number of patients who had a drug attributed to the subclass in their baseline medication list. “Adherent” indicates the number of patients who were found to be adherent, “% adherent” indicates the latter in percent.

ESM Table 5. Absolute numbers of events and incidence rates by adherence to drug classes

Endpoint	Drug	Adherence	n	N	Share (%)	n events	N events	Share events (%)	Patient-years	Median follow-up (years)	IR per 500 py	IR per 1000 py
cardioren	general adherence	any non-adherence	492	1125	43.7	116	241	23.6	2498.59	5.13	23.21	46.43
cardioren	general adherence	total adherence	633	1125	56.3	125	241	19.8	3186.58	5.06	19.61	39.23
cardioren	antihypertensive drugs	any non-adherence	213	1091	19.5	61	232	28.6	1093.43	5.22	27.89	55.79
cardioren	antihypertensive drugs	total adherence	878	1091	80.5	171	232	19.5	4434.77	5.09	19.28	38.56
cardioren	antiplatelet drugs	any non-adherence	8	81	9.9	5	28	62.5	35.72	4.58	69.99	139.97
cardioren	antiplatelet drugs	total adherence	73	81	90.1	23	28	31.5	360.51	5.10	31.90	63.80
cardioren	diuretics	any non-adherence	117	587	19.9	24	118	20.5	616.81	5.22	19.45	38.91
cardioren	diuretics	total adherence	470	587	80.1	94	118	20.0	2376.84	5.14	19.77	39.55
cardioren	glucose lowering drugs	any non-adherence	106	981	10.8	22	210	20.8	517.16	5.09	21.27	42.54
cardioren	glucose lowering drugs	total adherence	875	981	89.2	188	210	21.5	4445.80	5.10	21.14	42.29
cardioren	lipid lowering drugs	any non-adherence	223	762	29.3	42	168	18.8	1117.03	5.11	18.80	37.60
cardioren	lipid lowering drugs	total adherence	539	762	70.7	126	168	23.4	2732.50	5.08	23.06	46.11
kidney	general adherence	any non-adherence	492	1125	43.7	84	161	17.1	2498.59	5.13	16.81	33.62
kidney	general adherence	total adherence	633	1125	56.3	77	161	12.2	3186.58	5.06	12.08	24.16
kidney	antihypertensive drugs	any non-adherence	213	1091	19.5	49	154	23.0	1093.43	5.22	22.41	44.81
kidney	antihypertensive drugs	total adherence	878	1091	80.5	105	154	12.0	4434.77	5.09	11.84	23.68
kidney	antiplatelet drugs	any non-adherence	8	81	9.9	1	16	12.5	35.72	4.58	14.00	27.99
kidney	antiplatelet drugs	total adherence	73	81	90.1	15	16	20.6	360.51	5.10	20.80	41.61
kidney	diuretics	any non-adherence	117	587	19.9	18	78	15.4	616.81	5.22	14.59	29.18
kidney	diuretics	total adherence	470	587	80.1	60	78	12.8	2376.84	5.14	12.62	25.24
kidney	glucose lowering drugs	any non-adherence	106	981	10.8	13	137	12.3	517.16	5.09	12.57	25.14
kidney	glucose lowering drugs	total adherence	875	981	89.2	124	137	14.2	4445.80	5.10	13.95	27.89
kidney	lipid lowering drugs	any non-adherence	223	762	29.3	29	110	13.0	1117.03	5.11	12.98	25.96
kidney	lipid lowering drugs	total adherence	539	762	70.7	81	110	15.0	2732.50	5.08	14.82	29.64
cv	general adherence	any non-adherence	492	1125	43.7	40	94	8.1	2498.59	5.13	8.00	16.01
cv	general adherence	total adherence	633	1125	56.3	54	94	8.5	3186.58	5.06	8.47	16.95
cv	antihypertensive drugs	any non-adherence	213	1091	19.5	18	92	8.5	1093.43	5.22	8.23	16.46
cv	antihypertensive drugs	total adherence	878	1091	80.5	74	92	8.4	4434.77	5.09	8.34	16.69
cv	antiplatelet drugs	any non-adherence	8	81	9.9	5	13	62.5	35.72	4.58	69.99	139.97
cv	antiplatelet drugs	total adherence	73	81	90.1	8	13	11.0	360.51	5.10	11.10	22.19
cv	diuretics	any non-adherence	117	587	19.9	8	46	6.8	616.81	5.22	6.48	12.97
cv	diuretics	total adherence	470	587	80.1	38	46	8.1	2376.84	5.14	7.99	15.99
cv	glucose lowering drugs	any non-adherence	106	981	10.8	10	85	9.4	517.16	5.09	9.67	19.34
cv	glucose lowering drugs	total adherence	875	981	89.2	75	85	8.6	4445.80	5.10	8.43	16.87
cv	lipid lowering drugs	any non-adherence	223	762	29.3	16	66	7.2	1117.03	5.11	7.16	14.32
cv	lipid lowering drugs	total adherence	539	762	70.7	50	66	9.3	2732.50	5.08	9.15	18.30

ESM Table 5. “General adherence” describes adherence to all screened cardiometabolic drugs. “N” describes all patients on a specific drug class, “n” absolute numbers of adherent and non-adherent (totally and partially) patients and “share” the according percentage. “N events” describes all events, “n events” the events in adherent and non-adherent subgroups, respectively, and “Share events” the latter in percent. The table further contains the median follow-up, patients-years, and incidence rates per 500 and 1000 patient-years. Cardioren, cardiorenal composite endpoint; kidney, kidney composite endpoint; cv, cardiovascular composite endpoint; IR, incidence rate; py, patient-years.

ESM Table 6. Absolute numbers of specific cardiovascular and kidney events (components of composite endpoints) and incidence rates by adherence to drug classes

Endpoint (composite)	Endpoint (component)	Drug	Adherence	n	N	n events	N events	Share events (%)	Patient-years	IR per 1000 py
cv	cv death	antihypertensive drugs	any non-adherence	144	805	6	27	4.2	728.44	8.24
cv	cv death	antihypertensive drugs	total adherence	661	805	21	27	3.2	3327.45	6.31
cv	cv death	antiplatelet drugs	any non-adherence	8	81	3	5	37.5	35.72	83.98
cv	cv death	antiplatelet drugs	total adherence	73	81	2	5	2.7	360.51	5.55
cv	cv death	diuretics	any non-adherence	117	587	1	18	0.9	616.81	1.62
cv	cv death	diuretics	total adherence	470	587	17	18	3.6	2376.91	7.15
cv	cv death	glucose lowering drugs	any non-adherence	106	981	7	31	6.6	517.16	13.54
cv	cv death	glucose lowering drugs	total adherence	875	981	24	31	2.7	4445.88	5.40
cv	cv death	lipid lowering drugs	any non-adherence	223	762	7	24	3.1	1117.03	6.27
cv	cv death	lipid lowering drugs	total adherence	539	762	17	24	3.2	2732.58	6.22
cv	nfmi	antihypertensive drugs	any non-adherence	144	805	3	23	2.1	724.32	4.14
cv	nfmi	antihypertensive drugs	total adherence	661	805	20	23	3.0	3296.14	6.07
cv	nfmi	antiplatelet drugs	any non-adherence	8	81	2	4	25.0	34.65	57.72
cv	nfmi	antiplatelet drugs	total adherence	73	81	2	4	2.7	357.37	5.60
cv	nfmi	diuretics	any non-adherence	117	587	3	18	2.6	616.81	4.86
cv	nfmi	diuretics	total adherence	470	587	15	18	3.2	2349.90	6.38
cv	nfmi	glucose lowering drugs	any non-adherence	106	981	3	31	2.8	513.47	5.84
cv	nfmi	glucose lowering drugs	total adherence	875	981	28	31	3.2	4405.45	6.36
cv	nfmi	lipid lowering drugs	any non-adherence	223	762	8	27	3.6	1100.68	7.27
cv	nfmi	lipid lowering drugs	total adherence	539	762	19	27	3.5	2703.59	7.03
cv	nfs	antihypertensive drugs	any non-adherence	144	805	7	26	4.9	709.06	9.87
cv	nfs	antihypertensive drugs	total adherence	661	805	19	26	2.9	3275.27	5.80
cv	nfs	antiplatelet drugs	any non-adherence	8	81	1	5	12.5	35.72	27.99
cv	nfs	antiplatelet drugs	total adherence	73	81	4	5	5.5	351.58	11.38
cv	nfs	diuretics	any non-adherence	117	587	5	18	4.3	609.15	8.21
cv	nfs	diuretics	total adherence	470	587	13	18	2.8	2349.10	5.53
cv	nfs	glucose lowering drugs	any non-adherence	106	981	1	33	0.9	514.68	1.94
cv	nfs	glucose lowering drugs	total adherence	875	981	32	33	3.7	4360.44	7.34
cv	nfs	lipid lowering drugs	any non-adherence	223	762	7	25	3.1	1100.16	6.36
cv	nfs	lipid lowering drugs	total adherence	539	762	18	25	3.3	2676.20	6.73
kidney	albuminuria	antihypertensive drugs	any non-adherence	144	805	22	94	15.3	666.05	33.03
kidney	albuminuria	antihypertensive drugs	total adherence	661	805	72	94	10.9	3134.15	22.97
kidney	albuminuria	antiplatelet drugs	any non-adherence	8	81	1	12	12.5	32.73	30.55
kidney	albuminuria	antiplatelet drugs	total adherence	73	81	11	12	15.1	322.09	34.15
kidney	albuminuria	diuretics	any non-adherence	117	587	15	65	12.8	581.16	25.81
kidney	albuminuria	diuretics	total adherence	470	587	50	65	10.6	2256.34	22.16
kidney	albuminuria	glucose lowering drugs	any non-adherence	106	981	10	109	9.4	479.43	20.86
kidney	albuminuria	glucose lowering drugs	total adherence	875	981	99	109	11.3	4177.85	23.70
kidney	albuminuria	lipid lowering drugs	any non-adherence	223	762	20	85	9.0	1062.54	18.82
kidney	albuminuria	lipid lowering drugs	total adherence	539	762	65	85	12.1	2553.51	25.46
kidney	kidney death	antihypertensive drugs	any non-adherence	144	805	2	4	1.4	728.44	2.75
kidney	kidney death	antihypertensive drugs	total adherence	661	805	2	4	0.3	3327.45	0.60

kidney	kidney death	antiplatelet drugs	any non-adherence	8	81	0	0	0.0	35.72	0.00
kidney	kidney death	antiplatelet drugs	total adherence	73	81	0	0	0.0	360.51	0.00
kidney	kidney death	diuretics	any non-adherence	117	587	0	2	0.0	616.81	0.00
kidney	kidney death	diuretics	total adherence	470	587	2	2	0.4	2376.91	0.84
kidney	kidney death	glucose lowering drugs	any non-adherence	106	981	0	2	0.0	517.16	0.00
kidney	kidney death	glucose lowering drugs	total adherence	875	981	2	2	0.2	4445.88	0.45
kidney	kidney death	lipid lowering drugs	any non-adherence	223	762	0	2	0.0	1117.03	0.00
kidney	kidney death	lipid lowering drugs	total adherence	539	762	2	2	0.4	2732.58	0.73
kidney	krt	antihypertensive drugs	any non-adherence	144	805	5	11	3.5	728.44	6.86
kidney	krt	antihypertensive drugs	total adherence	661	805	6	11	0.9	3327.45	1.80
kidney	krt	antiplatelet drugs	any non-adherence	8	81	0	3	0.0	35.72	0.00
kidney	krt	antiplatelet drugs	total adherence	73	81	3	3	4.1	360.51	8.32
kidney	krt	diuretics	any non-adherence	117	587	1	7	0.9	616.81	1.62
kidney	krt	diuretics	total adherence	470	587	6	7	1.3	2376.91	2.52
kidney	krt	glucose lowering drugs	any non-adherence	106	981	0	8	0.0	517.16	0.00
kidney	krt	glucose lowering drugs	total adherence	875	981	8	8	0.9	4445.88	1.80
kidney	krt	lipid lowering drugs	any non-adherence	223	762	3	7	1.4	1117.03	2.69
kidney	krt	lipid lowering drugs	total adherence	539	762	4	7	0.7	2732.58	1.46
kidney	egfr	antihypertensive drugs	any non-adherence	144	805	8	23	5.6	710.07	11.27
kidney	egfr	antihypertensive drugs	total adherence	661	805	15	23	2.3	3290.25	4.56
kidney	egfr	antiplatelet drugs	any non-adherence	8	81	0	3	0.0	35.72	0.00
kidney	egfr	antiplatelet drugs	total adherence	73	81	3	3	4.1	353.47	8.49
kidney	egfr	diuretics	any non-adherence	117	587	2	8	1.7	610.84	3.27
kidney	egfr	diuretics	total adherence	470	587	6	8	1.3	2363.33	2.54
kidney	egfr	glucose lowering drugs	any non-adherence	106	981	3	26	2.8	510.61	5.88
kidney	egfr	glucose lowering drugs	total adherence	875	981	23	26	2.6	4391.09	5.24
kidney	egfr	lipid lowering drugs	any non-adherence	223	762	8	20	3.6	1099.45	7.28
kidney	egfr	lipid lowering drugs	total adherence	539	762	12	20	2.2	2704.98	4.44

ESM Table 6. "N" describes all analysed patients, "n" the absolute numbers of adherent and non-adherent (totally and partially) patients. "N events" describes all events, "n events" the events in adherent and non-adherent subgroups, respectively, and "Share events" the latter in percent. The table further contains the patients-years and incidence rates per 1000 patient-years. Cardioren, cardiorenal composite endpoint; kidney, kidney composite endpoint; cv, cardiovascular composite endpoint; cv death, death due to cardiovascular cause; nfmi, non-fatal myocardial infarction; nfs, non-fatal stroke; albuminuria, sustained progression of albuminuria from normal/mildly increased albuminuria (UACR < 30 mg/g creatinine) to moderately increased (UACR 30-300 mg/g creatinine) or severely increased albuminuria (UACR > 300 mg/g creatinine) or from moderately increased albuminuria to severely increased albuminuria including a > 30% increase in the mean UACR from baseline; kidney death, death from kidney failure; krt, initiation of kidney replacement therapy (dialysis or transplantation); egfr, sustained 40% reduction in eGFR (if baseline eGFR was > 60 ml/min/1,73m^2, eGFR had to additionally fall below 60 ml/min/1,73m^2), IR, incidence rate; py, patient-years; UACR, urinary albumin/creatinine ratio; eGFR, estimated glomerular filtration rate.

ESM Fig. 1. Comparison of target values by adherence to corresponding drug classes

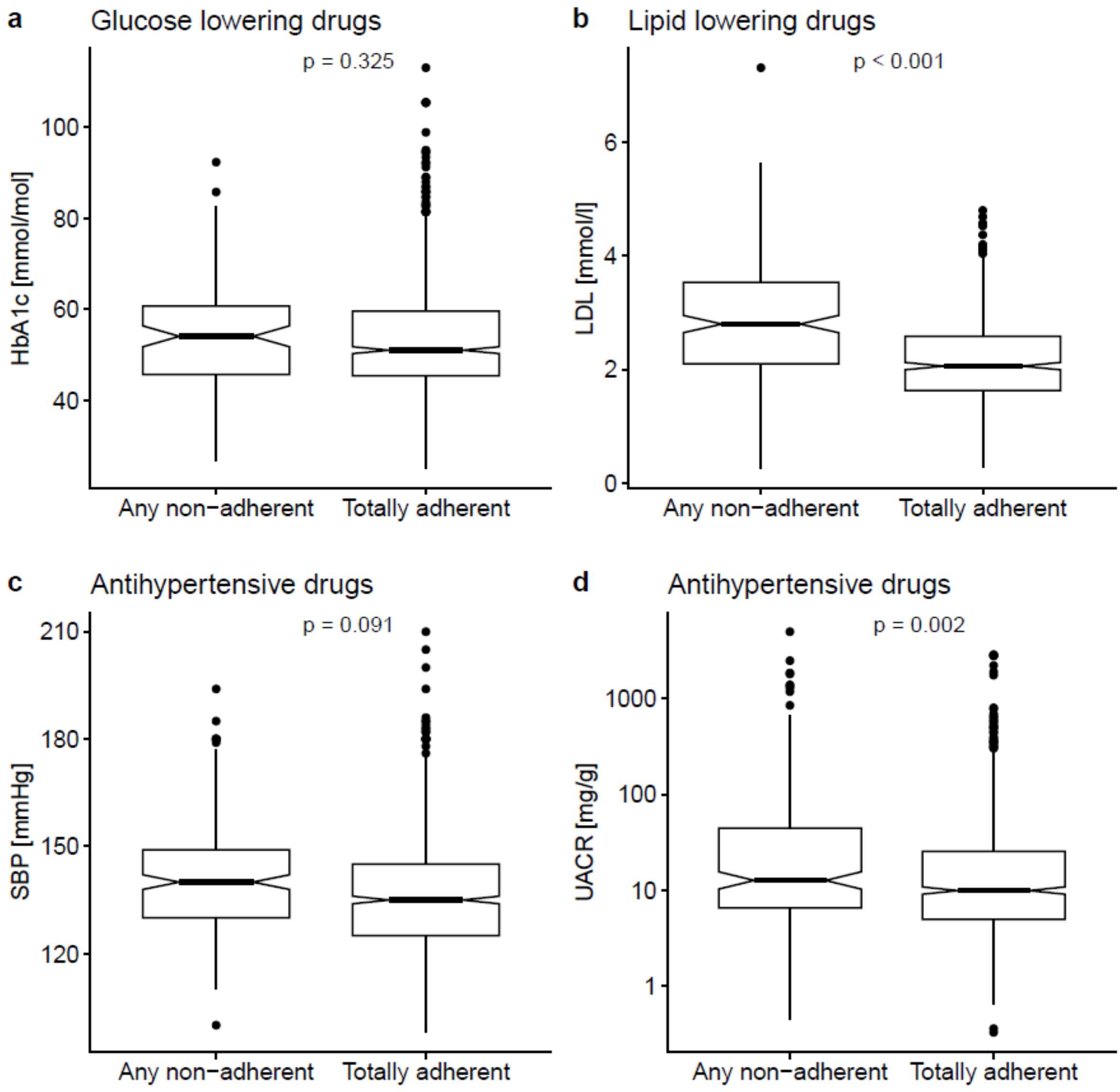

ESM Figure 1. Boxplots display the distributions of HbA_{1c}, LDL, SBP and UACR at baseline split by adherence to the corresponding drug classes. The lower and upper hinges correspond to the first and third quartiles (the 25th and 75th percentiles). The upper whisker extends from the hinge to the largest value no further than 1.5 * IQR from the hinge. The lower whisker extends from the hinge to the smallest value at most 1.5 * IQR of the hinge. Data beyond the end of the whiskers are called "outlying" points and are plotted individually. Notches around the median, in addition, indicate 95% CI. P-values for group differences were calculated using Wilcoxon–Mann–Whitney tests. HbA_{1c}, haemoglobin A1c; LDL, low-density lipoprotein; SBP, systolic blood pressure; UACR, urinary albumin/creatinine ratio.

ESM Fig. 2. Adherence to drug classes by gender

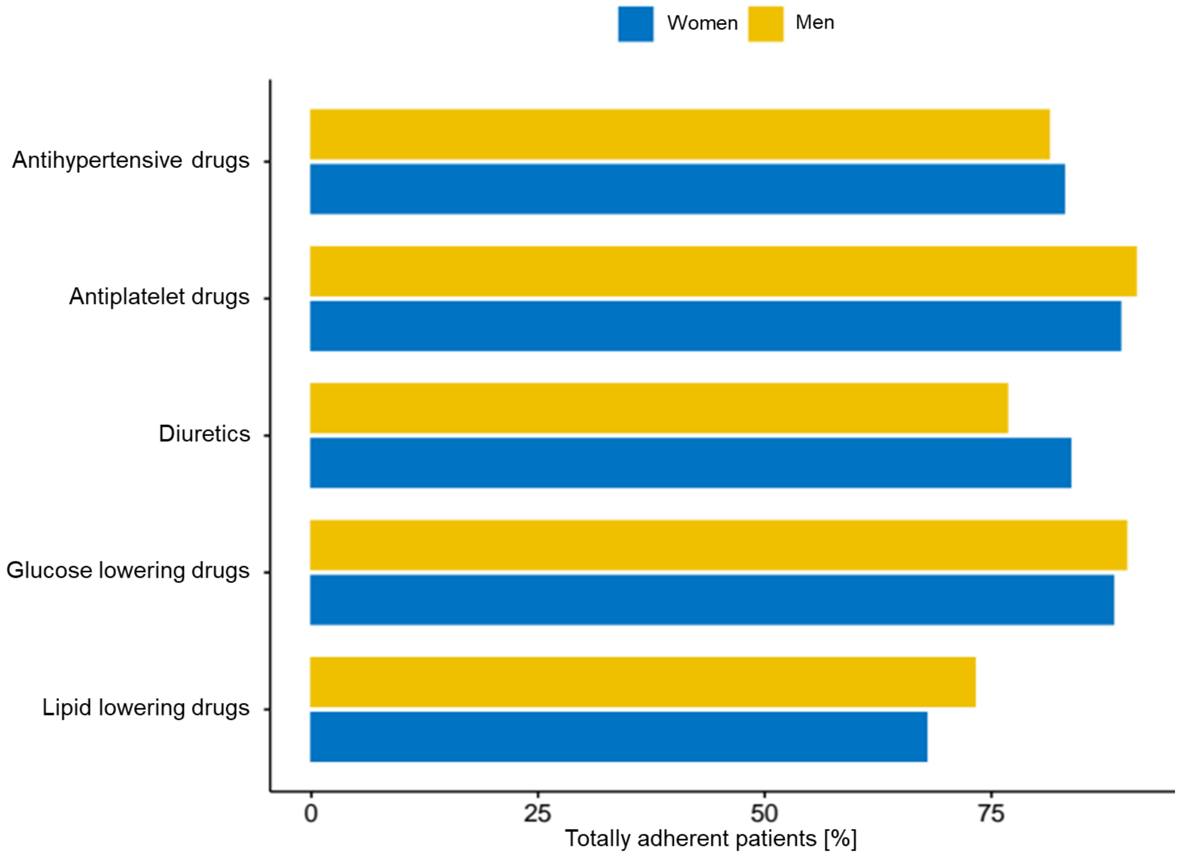

ESM Figure 2. Blue bars represent the percentages of totally adherent women and yellow bars the percentages of totally adherent men.
